# Wonderful Discovery

**Published:** 1842-03

**Authors:** 


*Wonderful Discovery.*—It will be seen from the following statement, which we extract from one of our daily newspapers, that *Dr. Jones,* of Baltimore, has made a discovery that is likely to throw his amalgum of mercury and silver, better known by the name of *"mineral cement,"* for filling teeth, into the shade.

"Dr. Jones, of St. Paul street, has shown us another tooth, the cavity of which is filling by ossification, or the growth of new bone; it is an upper grinding tooth having three roots, into two of which the decay had penetrated so as to make a large continuous cavity within the body; the cavity in one was large enough to admit a lump of cotton as large as a small pea, the other not quite so large; the new bone has filled both the roots, and a small portion of the body of the tooth."

The description of the tooth here given, it will be perceived, is that of one whose inner walls are totally devoid of life, so that the doctor must have indeed, possessed wonderful skill, to have resuscitated and endowed them with attributes that a living tooth does not possess. But, as he is becoming somewhat celebrated for making discoveries, or perhaps we ought to say, for having awarded to him, the authorship of discoveries made by others, we might be considered as wanting in proper credulity, were we to doubt his ability to impart to dead dental bone, recuperative powers. But seriously, we wonder if he understands the doctrine of the formation of dental bone? We very much question it, for if he does, he would at once perceive the utter impossibility of the filling up of a carious cavity in a tooth, and more especially that of one whose inner walls had been deprived of vitality, which is always the case with a tooth after the destruction of its lining membrane. That the editor of a newspaper should be deceived in a matter with which it is presumed he is wholly unacquainted, is not to be wondered, and in this case, we doubt not that the statement was founded more upon Dr. Jones' representation, than from a close physiological investigation of the supposed phenomenon.

